# Stability Study and Handling Recommendations for Multiresidue Pesticide Mixes under Diverse Storage Conditions for LC–MS/MS and GC–MS/MS

**DOI:** 10.1093/jaoacint/qsad096

**Published:** 2023-09-13

**Authors:** Landon A Wiest, Jana R Hepner, Jason E Fisher, Karen M Risha, John H Lidgett, Valerie N Ballarotto, Joseph D Konschnik

**Affiliations:** Restek Corporation, 110 Benner Circle, Bellefonte, PA 16823, United States; Restek Corporation, 110 Benner Circle, Bellefonte, PA 16823, United States; Restek Corporation, 110 Benner Circle, Bellefonte, PA 16823, United States; Restek Corporation, 110 Benner Circle, Bellefonte, PA 16823, United States; Restek Corporation, 110 Benner Circle, Bellefonte, PA 16823, United States; Restek Corporation, 110 Benner Circle, Bellefonte, PA 16823, United States; Restek Corporation, 110 Benner Circle, Bellefonte, PA 16823, United States

## Abstract

**Background:**

In response to the growing global need for pesticide residue testing, laboratories must develop versatile analytical methods and workflows to produce scientifically sound results. One of the many challenges faced by food chemists is acquiring suitable pesticide certified reference materials (CRMs) to calibrate analytical equipment, monitor method performance, and confirm the identity and concentration of hundreds of pesticide residues in food samples. CRM producers invest considerable resources to ensure the stability of their products.

**Objective:**

To present proper CRM handling and storage practices as guidance to ensure stability based on the results of several multiresidue pesticide stability studies.

**Methods:**

The open ampoule and combined multiresidue mix studies were conducted under controlled conditions. New ampoules containing multiresidue pesticide CRM mixtures were opened and compared to previously opened ampoules at multiple intervals while stored under freezing and refrigerated temperatures. Both LC- and GC-amenable pesticides (>200 residues) were combined and stored under typical laboratory conditions. Studies were performed with and without celery matrix.

**Results:**

The open ampoule study showed high levels of stability for all mixtures. All GC residues remained stable over the duration of the experiment. A week after opening LC multiresidue pesticide mixtures showed minor degradation. After combination of the multiresidue pesticide mixtures, degradation occurred rapidly for both the GC and LC mixtures.

**Conclusion:**

Multiresidue pesticide mixtures are stable as ampullated until they are opened. Once the contents of a kit were opened and combined, decreasing stability was observed over time. This was true for both the LC and GC kits. Working mixtures of CRMs for instrument calibration should be made daily.

**Highlights:**

This article shows a novel approach for measuring stability of CRM mixes. In-depth analysis of multiresidue pesticide mixtures and the stability that can be expected before and after mixing under typical storage conditions is described.

The world of multiresidue pesticide testing in food using GC–MS/MS and LC–MS/MS is rapidly growing. Where traditional methods had been used to identify and quantify tens of compounds, laboratories now routinely analyze hundreds of pesticides from different classes. While such comprehensive workflows have many benefits, they offer challenges to food safety chemists who are tasked with developing such testing methods. Among these challenges are chemical incompatibility and degradation (1) which are characteristic of many pesticides (2).

In addition to testing for legacy pesticides, which have existed for decades, food testing laboratories must also analyze for newer pesticides which have been designed to accomplish their intended purpose, then dissipate or degrade to a safe level prior to harvest (3–5). Agricultural chemicals in recent decades, unlike their predecessors, are designed this way to protect the environment and consumers from ingesting the same chemicals used to eliminate pests and weeds.

The term pesticide includes chemicals used in products, such as insecticides, fungicides, rodenticides, insect repellants, weed killers, and antimicrobials designed to prevent, destroy, repel, or reduce pests (6). In the United States, for example, the Environmental Protection Agency (EPA) legally regulates the pesticides that are used by growers to protect crops grown for human food and animal feed, and sets limits on the amounts of pesticides that may remain in or on foods marketed in the United States. These limits on pesticides left on foods are called “tolerances” in the United States (they are referred to as maximum residue limits [MRLs] in many other countries; 6). Before a pesticide may be marketed and used in the United States, the EPA evaluates the proposed pesticide thoroughly to ensure that it will not harm human health or the environment (7). In Europe, plant protection products cannot be placed on the market or used without prior authorization. A dual system is in place, under which the European Food Safety Authority (EFSA) evaluates active substances used in plant protection products and Member States evaluate and authorize the products at national level (8).

Although a benefit to growers and consumers, such chemicals pose a great challenge to analytical chemists who must test our food supply to ensure residues remaining after harvest are at levels that are safe for consumption. To meet this challenge the analytical testing community must understand to what extent, and how quickly these compounds degrade, knowing they are designed to do so (9). These chemicals must remain intact long enough to accurately measure them in our food supply.

Analytical chemists must be careful in handling these chemicals as they prepare reference material (RM) calibration solutions necessary to ensure delivery of accurate and precise chemical analysis results. Chemists will use such solutions to calibrate their laboratory equipment and verify their systems remain compliant with specified quality control (QC) criteria during routine testing. Analytical chemists may either formulate and prepare their own reference material solutions and mixtures or purchase them pre-mixed and packaged from commercial suppliers and producers as certified materials. Producers of reference materials must ensure certain minimum quality assurance (QA) requirements are met to certify their chemical standard solutions and guarantee they will consistently meet analytical chemists’ needs. They must also comply with and meet the rigorous quality system requirements should they decide to certify their solutions as certified reference materials (CRMs) under the ISO-IEC 17034–2017 standard (10). CRMs are defined in the standard as a reference material characterized by a metrologically valid procedure for one or more specified properties, accompanied by an RM certificate and issued by an authoritative body that provides the value of the specified property, its associated uncertainty, and a statement of metrological traceability.

When preparing CRM chemical solutions for pesticide analysis there are many important considerations. First, confirming the identity and purity of starting materials is paramount to ensuring subsequent steps produce an accurate solution. ISO-IEC 17034 requires this along with establishing and maintaining traceability to starting materials. Second, solutions must be carefully formulated to ensure the analytes contained in each solution are stable from the time of manufacture until the storage container is opened by the end-user. Well-defined handling, packaging, and storage procedures ensure their solutions remain stable until the expiry date stated on the reference material certificate and product label. Third, consistency among manufactured lots is also of significant importance to ensure standards produced are comparable and reproducible.

Understanding the compatibility of compounds, when multiple pesticides are combined into mixtures, requires knowledge, experience, and skill. It is imperative that the compatibility of multiple compounds in mixtures and their stability are verified using data from long- and short-term stability studies (2, 11–16). Such studies confirm formulation, packaging, and storage conditions meet the needs of their intended use.

Although reference material producers invest heavily in accounting for the above factors in supplying pesticide solutions and mixtures and guarantee the products they offer, such controls extend only until the container is opened by the end-user. Once a reference material product is received by a laboratory, the responsibility for proper storage, handling, and use rests on the user of such materials. Producers have no control over how their products are handled and used after delivery and when they are opened; however, they are required to include recommended storage conditions on their reference material certificates. Understanding this is crucial to ensuring a laboratory’s quality assurance program includes guidance and procedures to account for such practices. Knowing how to properly handle and store reference materials protects the laboratory’s investment since such materials can be expensive and impacts their operational efficiency and performance.

Stability and incompatibility concerns must also be considered after the container is opened in the laboratory and used to prepare solutions for calibration and QC. Prior to analysis in a testing laboratory, it is common to combine multiple solutions containing multiple pesticide compounds into larger multiresidue mixtures to expand the number of pesticides tested. Food safety testing laboratories now test for between 200 and 500 compounds routinely. Such comprehensive target analyte lists are necessary since many commodities are imported and exported globally and regulations for the use of agricultural chemicals vary among different nations. When chemists combine many pesticides into single multiresidue mixtures, stability and compatibility are not commonly understood, let alone determined and investigated.

Other research has preceded this work indicating this is not an entirely new issue in food testing. Maštovská and Lehotay published an evaluation of how various organic solvents impacted the stability of multiclass pesticides when analyzed by GC. Their work included 31 pesticides, six solvents, and their behavior when analyzed in zucchini, tomato, and grape commodities. The authors concluded the stability of certain pesticides improved when 0.1% (v/v) acetic acid was added to solutions prior to storage and subsequent analysis (11). Dorweiler et al. conducted a more comprehensive study where they evaluated the stability of 528 pesticides, metabolites, and contaminants in solvent over 6 months, and 1- and 2-year periods (12). Analytical techniques included both GC and LC–MS/MS. Studies such as these are most valuable in gaining understanding about the behavior of pesticides when mixed, stored, and used during routine food testing laboratory conditions.

In this publication, the authors will share results from three new multiresidue pesticide stability studies conducted by a reference material producer with the expectation of finding answers to questions often asked by its customers regarding the stability of its products during routine laboratory use. Restek’s GC and LC Multiresidue Pesticide Kits, each containing more than 200 pesticides packaged in 9 and 10 ampoules respectively, were the focus of these stability studies with the intention of answering such questions.

The first study measures the stability of each pesticide contained in each kit over a period of 31 days after opening. This study was conducted to gain knowledge regarding how long a pesticide CRM solution will remain stable after opening its package and storing under proper conditions between uses. The other two stability studies were designed to better understand what happens to pesticides when the individual multiresidue kit solutions are combined into a single multiresidue mix containing hundreds of compounds and used under routine laboratory conditions for food sample analysis over a specified period of time.

Sharing the results of their studies, the authors hope to educate and inform end-users about the proper handling and use of multiresidue pesticide mixtures and what to expect when they are combined for sample analysis. In addition to understanding such phenomena, the authors offer guidance enabling end-users to implement best practices in their laboratory operations, with the expectation of protecting their consumables investment and enhancing quality systems procedures.

## Experimental

### Chemicals and Supplies

LC and GC Multiresidue Pesticide Kits, quick, easy, cheap, effective, rugged, safe (QuEChERS) salts, dispersive solid phase extraction (dSPE) sorbents, Thomsen filter vials, Raptor ARC-18 and Allure C18 HPLC analytical columns, Raptor ARC-18 EXP guard cartridge, EXP guard holder, Ultra Shield in-line filter, Rtx-5 and Rxi-5ms GC columns, and Topaz GC liners were obtained from Restek Corp. (Bellefonte, PA, United States). Acetonitrile was obtained from Fisher Scientific (Hampton, NH, United States). Deionized water (18.3 MΩ/cm) came from a Barnstead water purification unit (Lake Balboa, CA, United States). Acetic acid (glacial), formic acid, and ammonium formate were obtained from Millipore Sigma (St. Louis, MO, United States). Celery was obtained from a local market and homogenized using a Blixer 3 Series D with 5 ½ quart capacity from Robot Coupe (Ridgeland, MS, United States).

### Open Ampoule Stability Study Design

The goal of this stability study was to conduct an experiment to gain a better understanding of how long the mixtures contained in each multiresidue kit ampoule remain stable after opening when stored properly.

**Figure 1.**
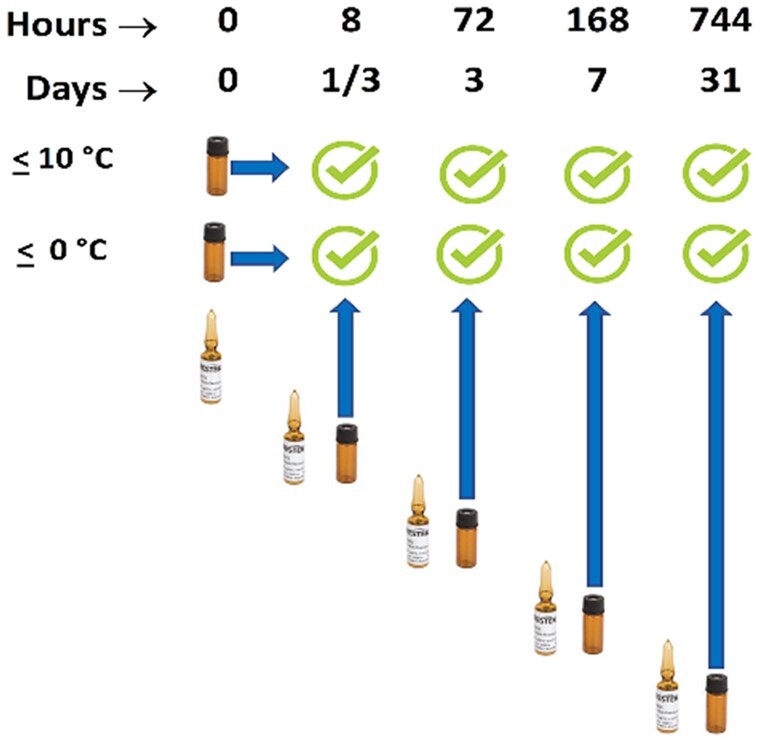
Schematic showing the experimental design of the ampoule stability study.

- *Instrumentation.—*For the open ampoule study, all LC samples were analyzed on a Shimadzu (Kyoto, Japan) Prominence XR equipped with a degasser, solvent pumps, auto injector, column oven, and photodiode array detector at wavelengths 220 and 254 nm. The GC samples were analyzed with an Agilent Technologies 7890 (Santa Clara, CA, United States) equipped with a flame ionization detector and autosampler.
- *Analysis.—*The study (Figure 1) commenced with opening an entire set of 10 ampoules from the LC kit and 9 ampoules from the GC kit (Table 1). A small portion of each ampoule was transferred to an autosampler vial and analyzed the same day by the appropriate technique. The remaining solution of each ampoule was split and transferred to two deactivated storage vials provided in the kit, then one vial was stored in a refrigerator (10 °C or colder) and the other in a freezer (0 °C or colder).

**Table 1.** Contents of the GC and LC Multiresidue Pesticide Kits

GC Multiresidue Pesticide Kit (each compound @ 100 μg/mL)	LC Multiresidue Pesticide Kit (each compound @ 100 μg/mL)
Ampoule #1 (16 organophosphorus compounds)	Ampoule #1 (13 organophosphorus compounds)
Azinphos ethyl (2642–71-9)	Acephate (30560–19-1)
Azinphos methyl (86–50-0)	Carbaryl (Sevin) (63–25–2)
Chlorpyrifos (2921–88-2)	Dicrotophos (141–66–2)
Chlorpyrifos methyl (5598–13-0)	Dimethoate (60–51–5)
Diazinon (333–41-5)	Dimethomorph (110488–70-5)
EPN (2104–64-5)	Isocarbophos (24353–61-5)
Fenitrothion (122–14–5)	Methamidophos (10265–92-6)
Isazophos (42509–80-8)	Mevinphos (7786–34-7)
Phosalone (2310–17-0)	Monocrotophos (6923–22-4)
Phosmet (732–11-6)	Omethoate (1113–02-6)
Pirimiphos ethyl (23505–41-1)	Temephos (Abate) (3383–96-8)
Pirimiphos methyl (29232–93-7)	Trichlorfon (Dylox) (52–68–6)
Pyraclofos (89784–60-1)	Vamidothion (Vamidoate) (2275–23-2)
Pyrazophos (13457–18-6)	
Pyridaphenthion (119–12-0)	Ampoule #2 (16 carbamate/uron compounds)
Quinalphos (13593–03-8)	Alanycarb (83130–01-2)
	Aldicarb (116–06-3)
Ampoule #2 (40 organochlorine compounds)	Aldicarb sulfone (1646–88-4)
Aldrin (309–00-2)	Aldicarb sulfoxide (1646–87-3)
α-BHC (319–84-6)	Benfuracarb (82560–54-1)
β-BHC (319–85-7)	Butocarboxim (34681–10-2)
δ-BHC (319–86-8)	Butoxycarboxim (34681–23-7)
γ-BHC (Lindane) (58–89–9)	Ethiofencarb (29973–13-5)
Chlorbenside (103–17–3)	Furathiocarb (65907–30-4)
*cis*-Chlordane (5103–71-9)	Methabenzthiazuron (18691–97-9)
*trans*-Chlordane (5103–74-2)	Methiocarb (2032–65-7)
Chlorfenson (Ovex) (80–33–1)	Methomyl (16752–77-5)
Chloroneb (2675–77-6)	Oxamyl (23135–22-0)
2,4'-DDD (53–19-0)	Tebuthiuron (34014–18-1)
4,4'-DDD (72–54–8)	Thidiazuron (51707–55-2)
2,4'-DDE (3424–82-6)	Thiophanate-methyl (23564–05-8)
4,4'-DDE (72–55–9)	
2,4'-DDT (789–02-6)	Ampoule #3 (38 carbamate/uron compounds)
4,4'-DDT (50–29–3)	Bendiocarb (22781–23-3)
4,4'-Dichlorobenzophenone (90–98–2)	Bifenazate (149877–41-8)
Dieldrin (60–57–1)	Carbofuran (1563–66-2)
Endosulfan I (959–98-8)	Chlorfluazuron (71422–67-8)
Endosulfan II (33213–65-9)	Chloroxuron (1982–47-4)
Endosulfan ether (3369–52-6)	Chlortoluron (15545–48-9)
Endosulfan sulfate (1031–07-8)	Cycluron (2163–69-1)
Endrin (72–20–8)	Diethofencarb (87130–20-9)
Endrin aldehyde (7421–93-4)	Diflubenzuron (35367–38-5)
Endrin ketone (53494–70-5)	Dioxacarb (6988–21-2)
Ethylan (Perthane) (72–56-0)	Diuron (330–54-1)
Fenson (80–38–6)	Fenobucarb (BPMC) (3766–81-2)
Heptachlor (76–44–8)	Fenoxycarb (72490–01-8)
Heptachlor epoxide (isomer B) (1024–57-3)	Fenuron (101–42–8)
Hexachlorobenzene (118–74–1)	Flufenoxuron (101463–69-8)
Isodrin (465–73-6)	Fluometuron (2164–17-2)
2,4'-Methoxychlor (30667–99-3)	Forchlorfenuron (68157–60-8)
4,4'-Methoxychlor olefin (2132–70-9)	Hexaflumuron (86479–06-3)
Mirex (2385–85-5)	3-Hydroxycarbofuran (16655–82-6)
*cis*-Nonachlor (5103–73-1)	Indoxacarb (173584–44-6)
*trans*-Nonachlor (39765–80-5)	Iprovalicarb (140923–17-7)
Pentachloroanisole (1825–21-4)	Isoprocarb (2631–40-5)
Pentachlorobenzene (608–93-5)	Isoproturon (34123–59-6)
Pentachlorothioanisole (1825–19-0)	Linuron (330–55-2)
Tetradifon (116–29-0)	Lufenuron (103055–07-8)
	Metobromuron (3060–89-7)
Ampoule #3 (25 organonitrogen compounds)	Monolinuron (1746–81-2)
100 µg/mL each in toluene–acetonitrile (99 + 1), 1 mL/ampoule	Neburon (555–37-3)
Benfluralin (1861–40-1)	Novaluron (116714–46-6)
Biphenyl (92–52–4)	Pirimicarb (23103–98-2)
Chlorothalonil (1897–45-6)	Promecarb (2631–37-0)
Dichlofluanid (1085–98-9)	Propham (122–42–9)
Dichloran (99–30–9)	Propoxur (Baygon) (114–26–1)
3,4-Dichloroaniline (95–76–1)	Pyraclostrobin (175013–18-0)
2,6-Dichlorobenzonitrile (Dichlobenil) (1194–65-6)	Siduron (1982–49-6)
Diphenylamine (122–39–4)	Teflubenzuron (83121–18-0)
Ethalfluralin (55283–68-6)	Thiobencarb (28249–77-6)
Fluchloralin (33245–39-5)	Triflumuron (64628–44-0)
Isopropalin (33820–53-0)	
Nitralin (4726–14-1)	Ampoule #4 (63 organonitrogen compounds)
Nitrofen (1836–75-5)	Abamectin (71751–41-2)
Oxyfluorfen (42874–03-3)	Acetamiprid (135410–20-7)
Pendimethalin (40487–42-1)	Ametryn (834–12-8)
Pentachloroaniline (527–20-8)	Amitraz (33089–61-1)
Pentachlorobenzonitrile (20925–85-3)	Azoxystrobin (131860–33-8)
Pentachloronitrobenzene (Quintozene) (82–68–8)	Benalaxyl (71626–11-4)
Prodiamine (29091–21-2)	Benzoximate (29104–30-1)
Profluralin (26399–36-0)	Boscalid (188425–85-6)
2,3,5,6-Tetrachloroaniline (3481–20-7)	Butafenacil (134605–64-4)
Tetrachloronitrobenzene (Tecnazene) (117–18-0)	Carbetamide (16118–49-3)
THPI (Tetrahydrophthalimide) (1469–48-3)	Carfentrazone ethyl (128639–02-1)
Tolylfluanid (731–27-1)	Chlorantraniliprole (500008–45-7)
Trifluralin (1582–09-8)	Clofentezine (74115–24-5)
	Cymoxanil (57966–95-7)
Ampoule #4 (28 organonitrogen compounds)	Cyprodinil (121552–61-2)
100 µg/mL each in toluene, 1 mL/ampoule	Cyromazine (66215–27-8)
Acetochlor (34256–82-1)	Dimoxystrobin (149961–52-4)
Alachlor (15972–60-8)	Dinotefuran (165252–70-0)
Allidochlor (93–71-0)	Doramectin (117704–25-3)
Clomazone (Command) (81777–89-1)	Eprinomectin (123997–26-2)
Cycloate (1134–23-2)	Famoxadon (131807–57-3)
Diallate (*cis* & *trans*) (2303–16-4)	Fenazaquin (120928–09-8)
Dimethachlor (50563–36-5)	Fenhexamid (126833–17-8)
Diphenamid (957–51-7)	Fenpyroximate (111812–58-9)
Fenpropathrin (39515–41-8)	Flonicamid (158062–67-0)
Fluquinconazole (136426–54-5)	Fluazinam** (79622–59-6)
Flutolanil (66332–96-5)	Fludioxonil (131341–86-1)
Linuron (330–55-2)	Fluoxastrobin (361377–29-9)
Metazachlor (67129–08-2)	Flutolanil (66332–96-5)
Methoxychlor (72–43–5)	Furalaxyl (57646–30-7)
Metolachlor (51218–45-2)	Halofenozide (112226–61-6)
N-(2,4-Dimethylphenyl)formamide (60397–77-5)	Imazalil (35554–44-0)
Norflurazon (27314–13-2)	Imidacloprid (138261–41-3)
Oxadiazon (19666–30-9)	Ivermectin (70288–86-7)
Pebulate (1114–71-2)	Kresoxim methyl (143390–89-0)
Pretilachlor (51218–49-6)	Mandipropamid (374726–62-2)
Prochloraz (67747–09-5)	Mepanipyrim (110235–47-7)
Propachlor (1918–16-7)	Mepronil (55814–41-0)
Propanil (709–98-8)	Metaflumizone (139968–49-3)
Propisochlor (86763–47-5)	Metalaxyl (57837–19-1)
Propyzamide (23950–58-5)	Methoxyfenozide (161050–58-4)
Pyridaben (96489–71-3)	Moxidectin (113507–06-5)
Tebufenpyrad (119168–77-3)	Myclobutanil (88671–89-0)
Triallate (2303–17-5)	Nitenpyram (120738–89-8)
	Oxadixyl (77732–09-3)
Ampoule #5 (34 organonitrogen compounds)	Picoxystrobin (117428–22-5)
Atrazine (1912–24-9)	Piperonyl butoxide (51–03-6)
Bupirimate (41483–43-6)	Prochloraz (67747–09-5)
Captafol (2425–06-1)	Prometon (1610–18-0)
Captan (133–06-2)	Pymetrozine (123312–89-0)
Chlorfenapyr (122453–73-0)	Pyracarbolid (24691–76-7)
Cyprodinil (121552–61-2)	Pyrimethanil (53112–28-0)
Etofenprox (80844–07-1)	Pyriproxyfen (95737–68-1)
Etridiazole (2593–15-9)	Quinoxyfen (124495–18-7)
Fenarimol (60168–88-9)	Rotenone (83–79–4)
Fipronil (120068–37-3)	Secbumeton (26259–45-0)
Fludioxonil (131341–86-1)	Spiroxamine (118134–30-8)
Fluridone (Sonar) (59756–60-4)	Tebufenozide (112410–23-8)
Flusilazole (85509–19-9)	Tebufenpyrad (119168–77-3)
Flutriafol (76674–21-0)	Terbumeton (33693–04-8)
Folpet (133–07-3)	Triadimefon (43121–43-3)
Hexazinone (Velpar) (51235–04-2)	Trifloxystrobin (141517–21-7)
Iprodione (36734–19-7)	Zoxamide (156052–68-5)
Lenacil (2164–08-1)	
MGK-264 (113–48-4)	Ampoule #5 (30 organonitrogen compounds)
Myclobutanil (88671–89-0)	Acibenzolar-S-methyl (135158–54-2)
Paclobutrazol (76738–62-0)	Bupirimate (41483–43-6)
Penconazole (66246–88-6)	Buprofezin (69327–76-0)
Procymidone (32809–16-8)	Carboxin (5234–68-4)
Propargite (2312–35-8)	Clethodim (99129–21-2)
Pyrimethanil (53112–28-0)	Clothianidin (210880–92-5)
Pyriproxyfen (95737–68-1)	Cyazofamid (120116–88-3)
Tebuconazole (107534–96-3)	Ethiprole (181587–01-9)
Terbacil (5902–51-2)	Ethofumesate (26225–79-6)
Terbuthylazine (5915–41-3)	Fenamidone (161326–34-7)
Triadimefon (43121–43-3)	Fipronil (120068–37-3)
Triadimenol (55219–65-3)	Flubendiamide (272451–65-7)
Tricyclazole (Beam) (41814–78-2)	Flufenacet (Fluthiamide) (142459–58-3)
Triflumizole (68694–11-1)	Hexythiazox (78587–05-0)
Vinclozolin (50471–44-8)	Mefenacet (73250–68-7)
	Mesotrione (104206–82-8)
Ampoule #6 (18 synthetic pyrethroid compounds)	Methoprotryne (841–06-5)
Acrinathrin (101007–06-1)	Metribuzin (21087–64-9)
Anthraquinone (84–65–1)	Prometryne (7287–19-6)
Bifenthrin (82657–04-3)	Propargite (2312–35-8)
Bioallethrin (584–79-2)	Prothioconazole (178928–70-6)
Cyfluthrin (68359–37-5)	Pyridaben (96489–71-3)
lambda-Cyhalothrin (91465–08-6)	*Simetryn (1014–70-6)*
Cypermethrin (52315–07-8)	Sulfentrazone (122836–35-5)
Deltamethrin (52918–63-5)	Terbutryn (886–50-0)
Fenvalerate (51630–58-1)	*Thiabendazole (*148–79–8*)*
Flucythrinate (70124–77-5)	Thiacloprid (111988–49-9)
tau-Fluvalinate (102851–06-9)	Thiamethoxam (153719–23-4)
*cis*-Permethrin (61949–76-6)	Thiofanox (39196–18-4)
*trans*-Permethrin (61949–77-7)	Tricyclazole (Beam) (41814–78-2)
Phenothrin (*cis* & *trans*) (26002–80-2)	
Resmethrin (10453–86-8)	Ampoule #6 (28 organonitrogen compounds)
Tefluthrin (79538–32-2)	Baycor (Bitertanol) (55179–31-2)
Tetramethrin (7696–12-0)	Bromuconazole (116255–48-2)
Transfluthrin (118712–89-3)	Cyproconazole (94361–06-5)
	Diclobutrazol (75736–33-3)
Ampoule #7 (10 herbicide methyl esters)	Difenoconazole (119446–68-3)
100 µg/mL each in toluene, 1 mL/ampoule	Diniconazole (83657–24-3)
Acequinocyl (57960–19-7)	Epoxiconazole (133855–98-8)
Bromopropylate (18181–80-1)	Etaconazole (60207–93-4)
Carfentrazone ethyl (128639–02-1)	Ethirimol (23947–60-6)
Chlorobenzilate (510–15-6)	Etoxazole (153233–91-1)
Chlorpropham (101–21–3)	Fenarimol (60168–88-9)
Chlozolinate (84332–86-5)	Fenbuconazole (114369–43-6)
DCPA methyl ester (Chlorthal-dimethyl) (1861–32-1)	Fluquinconazole (136426–54-5)
Fluazifop-*p*-butyl (79241–46-6)	Flusilazole (85509–19-9)
Metalaxyl (57837–19-1)	Flutriafol (76674–21-0)
2-Phenylphenol (90–43–7)	Fuberidazole (3878–19-1)
	Hexaconazole (79983–71-4)
Ampoule #8 (24 organophosphorus compounds)	Ipconazole (125225–28-7)
Bromfenvinfos-methyl (13104–21-7)	Metconazole (125116–23-6)
Bromfenvinphos (33399–00-7)	Nuarimol (63284–71-9)
Bromophos ethyl (4824–78-6)	Paclobutrazol (76738–62-0)
Bromophos methyl (2104–96-3)	Penconazole (66246–88-6)
Carbophenothion (786–19-6)	Propiconazole (Tilt) (60207–90-1)
Chlorfenvinphos (470–90-6)	Tebuconazole (107534–96-3)
Chlorthiophos (60238–56-4)	Tetraconazole (112281–77-3)
Coumaphos (56–72–4)	Triadimenol (55219–65-3)
Edifenphos (17109–49-8)	Triflumizole (68694–11-1)
Ethion (563–12-2)	Triticonazole (131983–72-7)
Fenamiphos (22224–92-6)	
Fenchlorphos (Ronnel) (299–84-3)	Ampoule #7 (7 organonitrogen compounds)
Fenthion (55–38–9)	Emamectin-benzoate (155569–91-8)
Iodofenphos (18181–70-9)	Fenpropimorph (67564–91-4)
Leptophos (21609–90-5)	Spirodiclofen (148477–71-8)
Malathion (121–75–5)	Spinosad (168316–95-8)
Methacrifos (62610–77-9)	Spirotetramat (203313–25-1)
Profenofos (41198–08-7)	Spinetoram (J&L) (935545–74-7)
Prothiofos (34643–46-4)	Spiromesifen (283594–90-1)
Sulfotepp (3689–24-5)	
Sulprofos (35400–43-2)	Ampoule #8 (1 organonitrogen compound)
Terbufos (13071–79-9)	Hydramethylnon (67485–29-4)
Tetrachlorvinphos (22248–79-9)	
Tolclofos-methyl (57018–04-9)	Ampoule #9 (7 carbamate/uron compounds)
	Aminocarb (2032–59-9)
Ampoule #9 (8 organophosphorus compounds)	Desmedipham (13684–56-5)
Disulfoton (298–04-4)	Formetanate HCL (23422–53-9)
Fonofos (944–22-9)	Mexacarbate (Zectran) (315–18-4)
Methyl parathion (298–00-0)	Monceren (Pencycuron) (66063–05-6)
Mevinphos (7786–34-7)	Phenmedipham (13684–63-4)
Parathion (ethyl parathion) (56–38–2)	Propamocarb free base (24579–73-5)
Phorate (298–02-2)	
Piperonyl butoxide (51–03-6)	Ampoule #10 (1 carbamate/uron compound)
Triazophos (24017–47-8)	Carbendazim (10605–21-7)

After 8 h, new ampoules were cracked open, transferred to an autosampler vial and analyzed by both techniques. The stored vials opened at the beginning of the study at hours/days = 0 were reanalyzed along with the new set of ampoules by each corresponding technique. The same experiment was repeated four more times after 72 h (3 days), 168 h (7 days), and 744 h (31 days), where new sealed samples from new kits pulled from inventory were tested against the original aliquots stored in both a refrigerator and freezer. Figure 1 illustrates a schematic of the open ampoule study.

**Figure 2.**
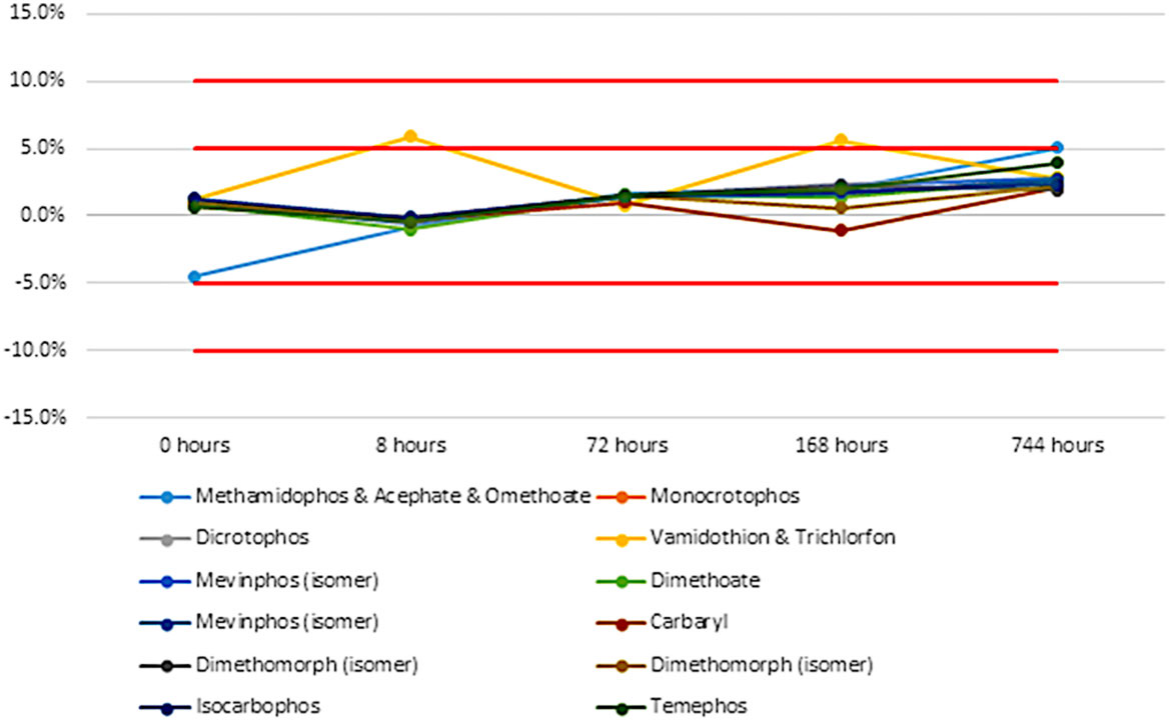
Chart showing percent difference mean at each analysis time with 5% and 10% acceptance criteria.

Five new LC and GC Multiresidue Pesticide Kits were taken from inventory and used over the course of the study. The contents of all vials were injected in triplicate and the average peak areas of each analyte were calculated. A percent difference was calculated using the mean of triplicate peak areas from the analysis of freshly opened ampoules as compared with the mean of the reanalyzed initial study vials. An RSD between triplicate injections of less than 5% was required for data to be included in the study. A 10% difference of means was assigned to the study as the criterion to determine which compounds remained stable and which failed. Any pesticide compound with a percent difference greater than 10% was considered degraded and failed the stability study. A percent difference of less than 10% passed. Figure 2 is an example of a chart used for displaying the percent difference mean of each pesticide from the initial value.

### Combined Multiresidue Mixtures: LC–MS/MS Study Design

The goal of this stability study was to determine how many LC Multiresidue Pesticide Kit compounds degrade and how many remain stable after combining the contents of all 10 ampoules into a single solution then storing them for analysis. To achieve this goal, each of the 10 ampoules, containing pesticides at the same concentration of 100 μg/mL, were opened and their contents transferred to the deactivated storage vial contained in the kit package. One hundred microliters from each storage vial were combined into a single vial with a 1.0 mL final volume. This produced a single multiresidue mixture containing all 204 compounds at a concentration of 10 μg/mL (Table 1). This solution was used to create a solvent calibration curve and subsequent dilutions for both a solvent analysis experiment and a celery matrix experiment. Table 2 describes specific details of the study design. Note the combined solvent pesticides mixture with a concentration of 10 μg/mL was diluted with a water–acetonitrile (9 + 1) solvent mixture for a final concentration of 1 μg/mL. This step was performed to match the extracted celery final concentration and create a suitable sample diluent close to the LC starting mobile phase conditions at injection.

**Table 2.** Combined multiresidue mixtures: LC–MS/MS study design

	204 Pesticides combined in solvent	204 Pesticides combined in matrix
Matrix	Solvent	Extracted celery
Solvent	Water–acetonitrile (9 + 1)	Water–acetonitrile (9 + 1)
Time	7 Days	9 Days
Concentration	1 ng/mL (ppb)	1 ng/mL (ppb)
Storage	(*1*) Autosampler^a^; (*2*) refrigerator^b^	Refrigerator^b^
Temperature	(*1*) 4 °C^a^; (*2*) 7 °C^b^	7 °C^b^

a Samples remained on the autosampler throughout the experiment and septa were not replaced.

b Samples were stored in the refrigerator between analyses. Septa were replaced daily.

- *Instrumentation.—*A Shimadzu Nexera UHPLC coupled to a Shimadzu LCMS 8060 was used for all LC–MS/MS analyses. Separations were performed with a Raptor ARC-18 2.7 µm, 100 mm × 2.1 mm HPLC column from Restek. To preserve the analytical column a guard cartridge (EXP Guard: Raptor ARC-18 2.7 µm, 5 mm × 2.1 mm) and in-line filter (UltraShield UHPLC PreColumn Filter) were used. The mobile phases for all LC–MS/MS experiments were 2 mM ammonium formate with 0.2% formic acid in water and 2 mM ammonium formate with 0.2% formic acid in methanol. The column was held at 50 °C with a flow rate of 0.4 mL/min. To expedite the chromatography and maintain narrow peaks, a gradient was used. The gradient began with 5% mobile phase B. The mobile phase gradient increased linearly to 60% B (0–2 min). The gradient slope changed (2–4 min) to 75% B and again (4–6 min) to 100% B and this was held for 1.5 min (6–7.5 min). After 7.5 min the column was allowed to reequilibrate for 2 min (7.51–9.5 min). All samples were in water–acetonitrile (9 + 1) solution. When matrix-matched, there was celery matrix contributed from the acetonitrile extract. All injection volumes were 5 µL. The analytes were ionized by electrospray ionization. For all LC–MS/MS conditions (retention times, precursor-to-product ion transitions, collision energies, etc.) *see*Supplemental Information, LCMSMS Conditions.
- *Sample preparation.—*Celery purchased from a local market was homogenized using a Robot Coup Blixer 3. After comminution, 15 g celery was fortified at 10 ng/g with all residues by adding 150 uL of a 1 μg/mL LC Multiresidue Pesticide Kit combined mixture which had been previously prepared that day. The fortified celery was then shaken for a couple of minutes to ensure uniform dispersion of the fortified pesticides in the slurry. Extraction was performed by adding 15 mL 1% acetic acid solution in acetonitrile (v/v) and AOAC Method **2007.01** QuEChERS salts. After centrifugation, 1 mL supernatant was added to a 2 mL dSPE vial containing magnesium sulfate, primary and secondary amine (PSA), and graphitized carbon black (GCB) dSPE for cleanup. After centrifugation, 100 µL of the 10 parts per billion (ppb) extract was diluted with 900 µL water and injected onto the ultra high-pressure liquid chromatograph (UHPLC).
- *Analysis.—*Test sample solutions were prepared in both solvent and celery matrix at a final concentration of 1 ng/mL in water–acetonitrile (9 + 1) solution and stored in vials. The solvent test sample vials were stored in a refrigerator at 7 °C over a 7-day period, however, these vials underwent a septum change and re-capping following analysis each day. Another set of solvent test sample vials were left on a Peltier-cooled autosampler tray, septum unchanged, at 4 °C throughout the study. The celery matrix vials were stored in a refrigerator (7 °C), and also underwent a septum change and re-capping after each day. Table 2 summarizes the stability study design.

Duplicate injections of each vial containing the solvent test sample solutions were made over a period of 7 days and duplicate injections of each vial containing celery matrix test sample solution were made over a 9-day period. The water–acetonitrile dilution was performed to closely match the LC mobile phase starting conditions as a best practice toward optimizing the analytical method of analysis. Calibration curves were run daily as a system suitability test. The calibration levels were 0.5, 1, 2, 5, and 10 ng/mL. The peak area of linuron-d6 from each day's calibration curve was used in the calculation of relative peak area ratios. This was done to eliminate bias in degradation results from the possible influence of instrument drift day to day. These study conditions were selected to simulate routine laboratory testing conditions at very low levels where pesticides’ stabilities are most challenged.

### Combined Multiresidue Mixtures: GC–MS/MS Study Design

This work was designed to complement the previous study but used pesticide residues amenable or specific to analysis via GC.

- *Instrumentation.—*A Thermo Fisher Scientific (Waltham, MA, United States) Trace 1310 GC coupled with a TSQ 8000 MS/MS was used for all analyses. Separation was performed on an Rxi-5MS 30 × 0.25 × 0.25 (Restek Corp., Bellefonte, PA, United States) using the following method: 90 °C (hold 1 min) to 330°C at 8.5 °C/min (hold 5 min). The inlet was held at 250 °C and 1 µL injections were performed in splitless mode with 0.5 min splitless time. For all GC–MS/MS conditions (retention times, precursor-to-product ion transitions, collision energies, etc.) *see*Supplemental Information, GCMSMS Conditions.
- *Sample preparation.—*On the same day, or the day before extraction, celery samples were homogenized until the sample matrix appeared uniform. Remaining portions of the celery sample were placed into storage containers for future use. When not used for analysis on the day of homogenization, the matrix was frozen and subsequently thawed for approximately 3 h prior to use.
  Prior to spiking 15 ± 0.2 g matrix was added to 50 mL centrifuge tubes. These were spiked with freshly prepared multiresidue pesticide mix at 100 ppb (final concentration). A total of 203 pesticide residues were present in this mixture. (Table 1) All were spiked with triphenyl phosphate at 20 ppb (final concentration) and then briefly shaken. After shaking, 15 mL acetonitrile with 1% acetic acid was added to the matrix and shaken for another minute. The new QuEChERS extraction salts were added to each sample. Each was shaken immediately for a few seconds after salt addition to prevent initial clumping of MgSO_4_. All samples were shaken for 1 min and centrifuged using a Q-sep 3000 centrifuge (Restek Corp.) at 3000*g*.
  Supernatant was removed after centrifugation. Further cleanup was performed by adding extract to the 15 mL dSPE sorbent vials containing MgSO_4_ and PSA. These were also shaken immediately after addition of the extract to prevent clumping and subsequently mixed on a vortex mixer. These test sample extracts were split into triplicate.
- *Analysis.—*Solvent calibrations were prepared from the same stock solutions at the same levels (in acetonitrile) as for matrix-matched samples. These test sample extracts were also split into triplicate.

One portion of the test samples was kept in a freezer, the second portion in the refrigerator, and the third portion was left on the autosampler rack. All test samples were recapped between analyses. Table 3 summarizes the design of this experiment.

**Table 3.** Combined multiresidue mixtures: GC–MS/MS study design^a^

	203 Pesticides combined in solvent	203 Pesticides combined in matrix
Matrix	Solvent	Extracted celery
Solvent	Acetonitrile	Acetonitrile
Time	17 Days	17 Days
Concentration	100 ng/mL (ppb)	100 ng/mL (ppb)
Storage between analyses^b^	(*1*) Refrigerator; (*2*) freezer; (*3*) autosampler	(*1*) Refrigerator; (*2*) freezer; (*3*) autosampler
Storage temperature	(*1*) 7 °C; (*2*) 4 °C; (*3*) room temperature	(*1*) 7 °C; (*2*) 4 °C; (*3*) room temperature

a Analysis occurred in triplicate.

b All samples were re-capped between analyses.

## Results and Discussion

### Open Ampoule Study

The GC Multiresidue Kit results, illustrated in Table 4, showed that no residues failed over the time period of the entire study. We should also note that storage conditions did not have a significant impact or cause failures for any of the residues in the GC kit.

**Table 4.** Open ampoule pesticides stability study: GC kit results^a^

	8 Hours		72 Hours		168 Hours		744 Hours	
	1/3 Day		3 Days		7 Days		31 Days	
Ampoule #	≤0 °C	≤10 °C	≤0 °C	≤10 °C	≤0 °C	≤10 °C	≤0 °C	≤10 °C
1	P	P	P	P	P	P	P	P
2	P	P	P	P	P	P	P	P
3	P	P	P	P	P	P	P	P
4	P	P	P	P	P	P	P	P
5	P	P	P	P	P	P	P	P
6	P	P	P	P	P	P	P	P
7	P	P	P	P	P	P	P	P
8	P	P	P	P	P	P	P	P
9	P	P	P	P	P	P	P	P

a Criteria: ±10% of label concentration; P = Pass <±10%; F = Fail >±10%.

Results for the LC Multiresidue Kit, shown in Table 5, showed similar results. The tests were performed identically, however, there were four failures. In the second ampoule, containing carbamates and urons (i.e., phenylureas), benfuracarb (CAS# 82560–54-1) failed with a 11.5% variance, but only in the freezer-stored ampoule after 31 days. Isoprocarb (CAS# 2631–40-5), from the third ampoule failed under both refrigerator (10.2%) and freezer conditions (12.1%). This was also a carbamate and uron mixture. The fifth ampoule, containing organonitrogen pesticides, had two failing residues. Simetryn (CAS# 1014–70-6) failed after 7 days (19.2%) at 0°C and thiabendazole (CAS# 148–79-8) also failed after 7 days (10.1%) at 0°C.

**Table 5.** Open ampoule pesticides stability study: LC kit results^a^

	8 Hours		72 Hours		168 Hours		744 Hours	
	1/3 Day		3 Days		7 Days		31 Days	
Ampoule #	≤0 °C	≤10 °C	≤0 °C	≤10 °C	≤0 °C	≤10 °C	≤0 °C	≤10 °C
1	P	P	P	P	P	P	P	P
2	P	P	P	P	P	P	F	P
3	P	P	P	P	F	F	P	P
4	P	P	P	P	P	P	P	P
5	P	P	P	P	F	P	P	P
6	P	P	P	P	P	P	P	P
7	P	P	P	P	P	P	P	P
8	P	P	P	P	P	P	P	P
9	P	P	P	P	P	P	P	P
10	P	P	P	P	P	P	P	P

a Criteria: ±10% of label concentration; P = Pass <±10%; F = Fail >±10%.

### Open Ampoule Discussion

The vast majority of the pesticide mixes in the two multiresidue kits remained stable for the entire 31-day study period after opening ampoules and properly storing them for reuse. Three of the four failures with the LC kit were minimal and occurred during the 7-day test cycle within two ampoules. Two of the three compounds, isoprocarb and thiabendazole, failed just outside of the 10% variance error bar and were interpreted as analytical error since both passed during the 31 day cycle. Simetryn failed at 19.2% and, although a much higher percentage than the others on that same test cycle, was accounted for as an outlier since this compound passed within 10% error during the 31-day measurement. Refrigerator storage appeared to be slightly better for the stability of the residues, as the LC kit saw three of its four failures come from freezer storage. Results from this study indicate when properly handled and stored the multiresidue kit ampoules remained stable for 1 month, suggesting suitable stability for most routine testing laboratory practices.

### Combined Multiresidue Mixtures: LC–MS/MS Study

This experiment was designed to show the degradation of a matrix-matched calibration set reused for calibrating an instrument daily. Therefore, the test samples were in diluent, ready for HPLC analysis. The concentration for the calibration curve was from 0.5–10 ppb, and the test samples were at 1 ppb concentration.

Multiple test samples were made for each experiment. Each day a 5-level calibration curve was run before and after the test samples. Linuron-d6 was chosen to calculate all area ratios, as it was the most stable deuterated compound. The 5-level calibration curve was used to quantify the test sample mixes (*n* = 3). Average values were used to verify the signal intensity/concentration each day to ensure system suitability and stability. The data from each subsequent day were compared to the initial set of measurements. Signals that decreased by more than 20% were identified as failing.

**Figure 3.**
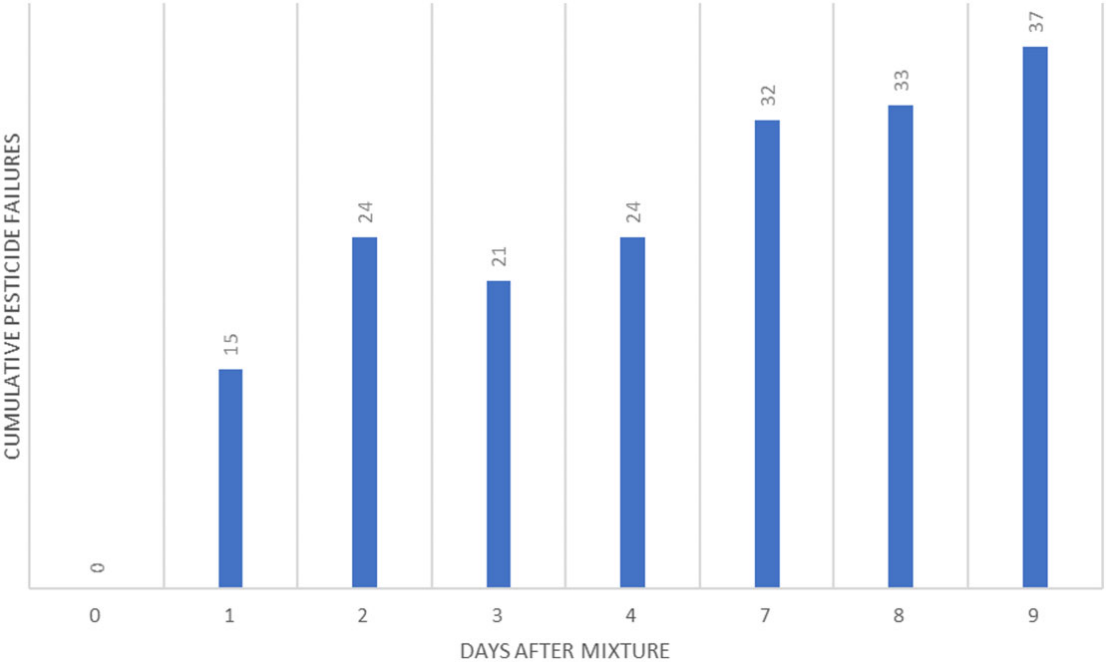
Number of pesticide residues from the LC Multiresidue Pesticide Kit that decreased by >20% from their initial concentration after being stored in water–acetonitrile (9 + 1) in celery matrix (1 ppb concentration) on a Peltier-cooled autosampler (4 °C). After analysis, caps were replaced and samples were placed in a 7 °C fridge. Area ratios were created with linuron-d6 as the internal standard.

Multiple experiments were performed. For the first experiment, all samples were kept on the Peltier cooled autosampler at 4 °C. Analytes were in water–acetonitrile (9 +1) with celery matrix at 1 ppb concentration. The amount of celery matrix present was equivalent to the amount that would be present during a real sample extraction. After a week of analyses, a greater than 20% decrease in concentration for 37 residues was observed (Figure 3).

**Figure 4.**
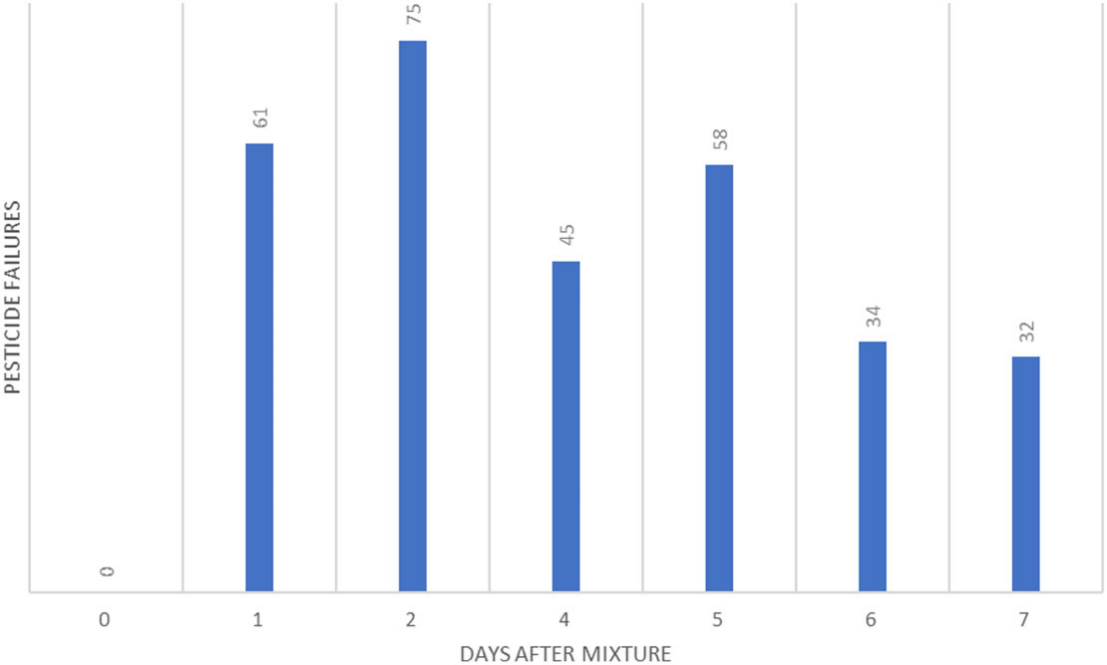
Number of pesticide residues from the LC Multiresidue Pesticide Kit that decreased by >20% from their initial concentration after being stored in water–acetonitrile (9 + 1). The mixture was only stored on a Peltier-cooled autosampler (4 °C). Area ratios were created using a linuron-d6 internal standard.

The next experiment (Figure 4) was designed to show similar laboratory practices but without celery matrix. All of the samples were kept on the Peltier-cooled autosampler (4 °C) throughout the duration of the weeklong experiment and the septa were never replaced.

**Figure 5.**
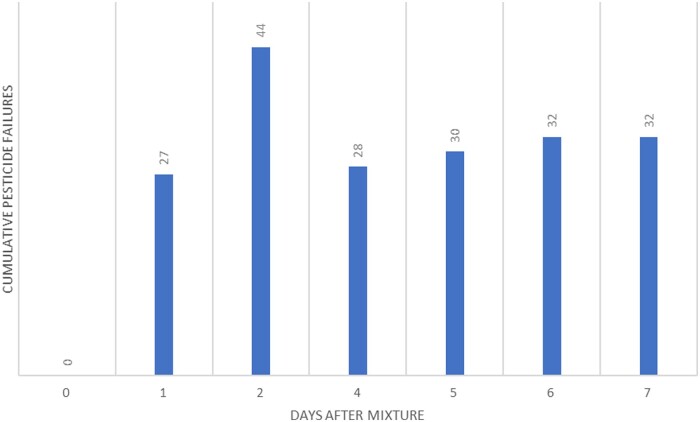
Number of pesticide residues from the LC Multiresidue Pesticide Kit that decreased by >20% from their initial concentration after being stored in water–acetonitrile (9 + 1). The mixture was stored on a Peltier-cooled autosampler (4 °C) during the day and septa were replaced daily with overnight storage in a refrigerator (7 °C). Area ratios were created with linuron-d6 as the internal standard.

The final experiment (Figure 5) was performed without matrix, but the samples were stored overnight in a refrigerator and septa were replaced.

### Combined Multiresidue Mixtures: LC–MS/MS Study Discussion

The data here indicates some trends one might not expect. Data obtained from Days 1 and 2 showed increasing pesticide residue degradation, as anticipated. On Days 3 and 4, we expected to see the trend continue, but this did not appear to be the case for these studies. Upon further inspection we found that some pesticides had failures near the 20% limit. On days where they failed, they failed slightly, and on days where they passed, they passed by a similarly slim margin. Day-to-day instrument variability also contributes to these borderline cases. Internal standards also played a role. Linuron-d6 was the most stable internal standard, but it degraded by an average of 17% over the course of the study. As degradation occurs at a different rate for each pesticide, the area ratios will be affected resulting in the observed stability trends. Matrix also contributes to analyte variability (17). In our experiment with celery matrix, the matrix appears to have either preserved the analytes or enhanced their signal (18, 19).

Diluents play a crucial role in analyte stability. After sample preparation extracts are stored in acetonitrile, which is typical for QuEChERS extractions. Acetonitrile is also a typical HPLC solvent/mobile phase, however in reversed-phase separations, which are the common separation method employed for pesticides analyses, acetonitrile is a strong solvent and must be diluted with water. The addition of water creates a very reactive environment for the pesticides, many of which are designed to be degraded by aqueous conditions. Accordingly, as standards degrade when stored in HPLC-suitable diluents, incoming samples will have biased data even though calibration curves may still appear linear.

With all these variables at play, it is critical that calibration standards be made fresh daily from properly stored standards and that analysis of incoming samples be done with freshly made calibration standards. Table 6 shows the pesticides that lost more than 50% signal over the course of the experiments. The list of compounds is cumulative day by day over the course of the study. Because of the reactive nature of the LC study storage conditions, a more extensive study was conducted with a GC Multiresidue Pesticide Kit where samples and standards were made without water.

**Table 6.** Pesticides that lost >50% initial signal after n days^a^

Never detected	1 Day	2 Days	3 Days	4 Days	7 Days	8 Days
Moxidectin	Thiophanate-methyl	Carboxin	Triflumizole	Hexythiazox	Butocarboxim	Propoxur
Mesotrione	Bifenazate	Diazinon-d10	Novaluron-H	Ivermectin	Aldicarb	Atrazine-d5
Chlorfluazuron	Prothioconazole	Fluazinam	Clethodim Isomer 1		Ethiofencarb	Pyracarbolid
Benfuracarb	hydramethylnon	Temephos	Pyriproxyfen		Chlorotoluron	Desmedipham
	Hexaflumuron-H	Lefenuron	Spirodiclofen		Isocarbophos	Fenamidone
	Metaflumizone	Chlorfluazuron			Fludioxonil	Thiofanox
	Fluazinam				Fipronil	Flubendiamide
	Flufenoxuron				Famoxadone	Difenoconazole
	Propargite				Triflumuron	Ipconazole
	Etoxazole				Indoxacarb	Emamectin B1b
	Spiromesifen+H				Teflubenzuron	
	Fenpyroximate				Alanycarb	
	Pyridaben				Quinoxyfen	
	Fenazaquin				Abamectin B1b	
					Eprinomectin	
					Doramectin	

a Compounds listed are cumulative over the duration of the study.

### Combined Multiresidue Mixtures: GC–MS/MS Study

The data from the first analysis were set as 100% and analysis from each following experiment were compared to the initial measurements. Signals that decreased to below 80% were identified as failing.

On Day 0, mixtures from all nine ampoules of the GC Multiresidue Pesticide Kit were prepared. These ampoules contained between 8 and 40 pesticides in each mixture. All mixtures were diluted in toluene and were formulated to be stable before opening and mixing.

Two stock solutions were made containing all nine ampoules mixed from the GC Multiresidue Pesticide Kit. One stock mixture was diluted in neat acetonitrile and the other was diluted in acetonitrile containing celery matrix. Each pesticide concentration was 100 ppb. After initial analysis, both stocks were split into three aliquots. The first aliquot was stored in a freezer. The second was stored under refrigerated conditions. The third was stored on an autosampler. Samples were spiked with the internal standard, triphenyl phosphate, and the samples were analyzed the first day, time (t) = 0.

**Figure 6.**
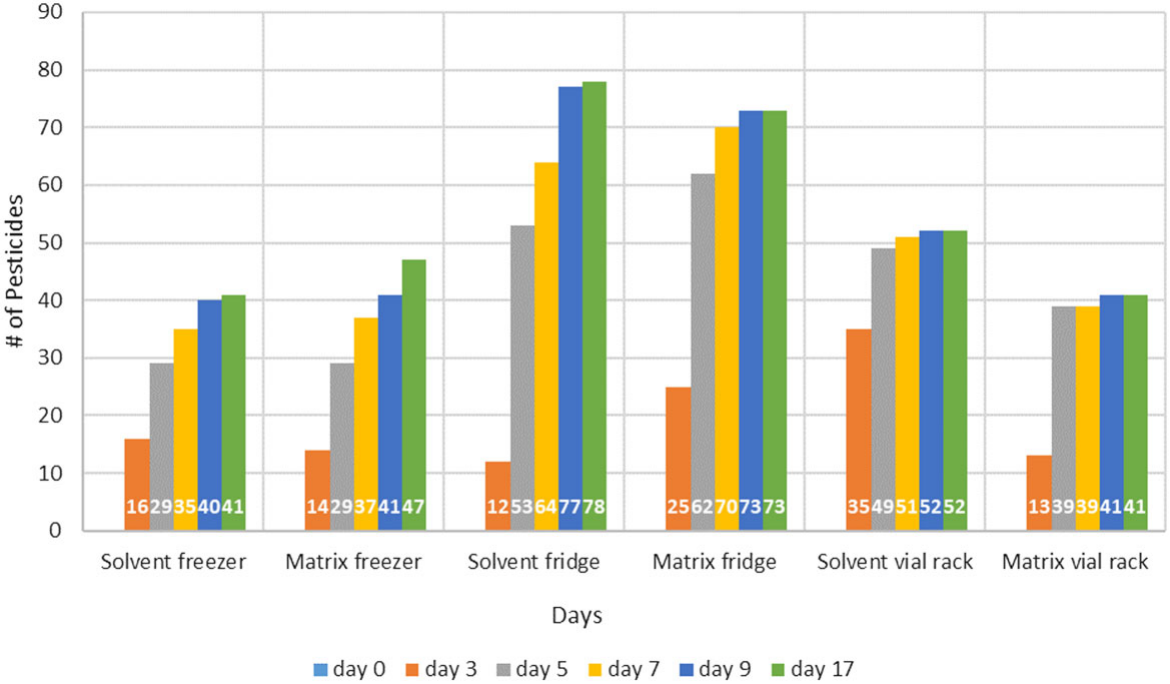
Number of pesticide residues from a GC Multiresidue Pesticide Kit that saw a decrease of >20% from the initial concentration after being stored in acetonitrile in celery matrix (100 ppb concentration) at the given conditions.

Samples were reanalyzed every 2–3 days up to 9 days and then on Day 17 (Figure 6). Samples were recapped after each analysis to prevent evaporation losses and septa contamination.

### Combined Multiresidue Mixtures: GC–MS/MS Discussion

Due to long instrument cycle times of approximately 40 min, the shortest time between analyses was every other day. The second analysis on Day 3 indicated that more than 6% of pesticide residues lost 20% or more of their signal. On average, there was not much difference between solvent and matrix samples. The exception was the solvent sample on the vial rack, where 35 residues lost more than 20% signal during the second analysis, however, this trend did not continue. Across the board, most pesticides with signal loss were observed in the samples kept in the fridge (78 and 73, solvent and matrix, respectively) and the least number was observed in the freezer (41, solvent samples) and on the vial rack (41, matrix; Figure 6).

This shows that no matter how the samples are stored, we observe a significant signal loss within a few days even when the samples are kept in the freezer. Table 7 shows the specific pesticide residues that lost more than 50% signal over the course of the experiments.

**Table 7.** Pesticides that lost >50% initial signal after n days after being stored on vial rack in acetonitrile with matrix, 100 ppb concentration^a^

3 Days	5 Days	7 Days	9 Days	17 Days
Acequinocyl	Aldrin	None	Captan	None
Alachlor	Atrazine		Profenofos	
Captafol	Azinphos-methyl			
Flusilazole	Bromophos methyl			
Nitrofen	Bromopropylate			
Nonachlor, *cis*-	Chlordane, *cis*-			
Prochloraz	Chlorothalonil			
Pyraclofos	DDT, o, p'-			
Resmethrin	Diallate			
Tebufenpyrad	Dieldrin			
Terbacil	Diphenamid			
Tetradifon	Fenamiphos			
Triadimenol	Fenarimol			
	Heptachlor			
	Heptachlor epoxide			
	Iodofenfos			
	Leptophos			
	Mirex			
	Norflurazon			
	Penconazole			
	Pentachloroaniline			
	Pretilachlor			
	Pyrazophos			
	Pyriproxyfen			
	Tricyclazole			

a Compounds listed are cumulative over the duration of the study.

### General Handling Recommendations

The open ampoule stability study indicated that when proper storage conditions are followed all of the pesticides contained in ampullated mixes, with few exceptions, remained stable for 31 days when stored in deactivated storage vials independent of refrigerator, or freezer temperatures. Internal stability studies have indicated that using deactivated storage vials will prolong analyte stability (10, 20–22). Deactivated vials prevent loss due to surface adsorption and decomposition by preventing interaction between pesticides and exposed silanol groups on glass vial surfaces. The authors recommend following CRM producer handling instructions which should be included in their Reference Material Certificate to get the best possible performance when using their products. Based on these studies, which were performed in the producer’s laboratories with their personnel, equipment, and systems, the authors concluded the ampullated mixes can be stored for around 31 days once opened.

### Faster Workflows Reduce Risk of Degradation

In addition to the above recommendations, other strategies may be considered when ampullated mixes are combined into multiresidue mixtures then used for sample processing under routine laboratory conditions. Pesticide residue mixtures react with each other, matrix, and mobile phase. Accordingly, reducing the amount of time to prepare and analyze samples will minimize the risk of sample degradation during handling and processing. Faster sample throughput, resulting from automated sample preparation techniques, can reduce the overall time it takes to prepare samples prior to analysis (23). Tandem sample preparation and analysis techniques such as in-line sample preparation (ILSP) are other strategies to increase workflow speeds and decrease the amount of time prepared samples wait for analysis (23). Other strategies may also be employed to reduce sample preparation time which may include eliminating cleanup and filtration steps and replacing them with a dilute-and-shoot step.

It is common practice to load multiple samples onto autosampler trays waiting for sample analysis to be completed. Extended amounts of time spent on the autosampler can lead to sample and standard degradation. With HPLC workflows this can be less of a problem than with GC workflows, as HPLC runtimes are usually on the order of 10–15 min for multiresidue pesticides. These faster runtimes allow for 100, or more, samples to be analyzed within a 24-hour period thereby minimizing the risk of degradation.

In the case of GC–MS/MS, and noted earlier, runtimes are typically much longer increasing the risk of multiresidue pesticide mixture degradation. There are multiple strategies that can be employed to increase sample throughput in GC. Using hydrogen as a carrier gas can speed up analysis time, as the optimal linear velocity of hydrogen is faster than carrier gases like nitrogen or helium (24–26). It is possible to simply increase the flow rate, but running above the optimum linear velocity promotes band broadening. Narrower and shorter columns can be employed as well to decrease runtime, but these columns can foul more easily when exposed to complex food matrixes, requiring more frequent trimming and maintenance. Backflushing is another method that decreases analysis time without reducing resolution, (27–30) however, it requires instrumentation changes and complex software. If resolution between analyte signals is not critical, other methods can be used. Trading resolution for faster analysis times may be acceptable when analysis is performed with a mass spectrometer. Another solution is using a shorter column with a wider diameter, where the analytical column is connected to the inlet through a guard column with a restrictor, allowing for near-vacuum in the analytical column and stable head pressure in the inlet. This is sometimes called vacuum gas chromatography, or low-pressure gas chromatography (LPGC; 31–33).

In addition, and also based on both the LC–MS/MS and GC–MS/MS combined multiresidue studies, the authors recommend that daily instrument calibration curve mixtures be prepared fresh daily. Since the number of pesticides which degraded within 1 or more days was significant such guidance seems appropriate to minimize the risk of an inaccurate instrument calibration prior to analysis. Along with this handling and usage recommendation the authors also recommend each laboratory conduct their own internal stability studies using their personnel, equipment, and systems since their operational goals may be different and some of the pesticides which degraded rapidly in the studies presented in this body of research may not be of interest to them, or their clients.

## Conclusions

These three stability studies demonstrate the stability of pesticide mixtures under various conditions representative of a routine food testing laboratory. Considering pesticide mixture stability when performing multiresidue pesticide analysis is crucial. The authors have demonstrated that ampullated pesticide mixtures are quite stable, when stored as recommended under both refrigerator and freezer storage temperatures. However, once these mixtures were combined they were no longer stable for multiple days. As demonstrated by the LC study, degradation occurs with several pesticides under all tested conditions. The GC study also showed degradation after 3 days and more pesticides continued to lose <20% signal over the duration of the experiment. Our recommendation to ensure the best data integrity is to prepare calibration standards daily when performing multiresidue pesticide analysis. Samples should also be processed quickly, preferably on the same day as preparation, as mixtures of pesticides in any matrix degrade over time if not analyzed in a timely manner. There are also various approaches, including LPGC and ILSP, to reduce analysis times to prevent pesticide degradation which may compromise data and results integrity.

The authors suggest future research be conducted to study not only pesticide degradation, but also remedies to improve stability in routine laboratory workflows. These include the following:

Comparison between deactivated storage vials versus non-deactivated storage vials to confirm whether there is a statistically significant difference between the two to improve pesticide stability over time following sample preparation and prior to analysis.
Addition of preservatives such as weak acids in LC final extracts to enhance stability prior to analysis.
Eliminating water in LC final extracts to enhance pesticide stability prior to analysis.

## Supplementary Material

qsad096_Supplementary_Data
